# The P2X7 Receptor Supports Both Life and Death in Fibrogenic Pancreatic Stellate Cells

**DOI:** 10.1371/journal.pone.0051164

**Published:** 2012-12-17

**Authors:** Kristian A. Haanes, Albrecht Schwab, Ivana Novak

**Affiliations:** 1 Department of Biology, University of Copenhagen, Copenhagen, Denmark; 2 Institut für Physiologie II, Universität Münster, Münster, Germany; Osaka University Graduate School of Medicine, Japan

## Abstract

The pancreatic stellate cells (PSCs) have complex roles in pancreas, including tissue repair and fibrosis. PSCs surround ATP releasing exocrine cells, but little is known about purinergic receptors and their function in PSCs. Our aim was to resolve whether PSCs express the multifunctional P2X7 receptor and elucidate how it regulates PSC viability. The number of PSCs isolated from wild type (WT) mice was 50% higher than those from the Pfizer P2X7 receptor knock out (KO) mice. The P2X7 receptor protein and mRNA of all known isoforms were expressed in WT PSCs, while KO PSCs only expressed truncated versions of the receptor. In culture, the proliferation rate of the KO PSCs was significantly lower. Inclusion of apyrase reduced the proliferation rate in both WT and KO PSCs, indicating importance of endogenous ATP. Exogenous ATP had a two-sided effect. Proliferation of both WT and KO cells was stimulated with ATP in a concentration-dependent manner with a maximum effect at 100 µM. At high ATP concentration (5 mM), WT PSCs, but not the KO PSCs died. The intracellular Ca^2+^ signals and proliferation rate induced by micromolar ATP concentrations were inhibited by the allosteric P2X7 receptor inhibitor az10606120. The P2X7 receptor-pore inhibitor A438079 partially prevented cell death induced by millimolar ATP concentrations. This study shows that ATP and P2X7 receptors are important regulators of PSC proliferation and death, and therefore might be potential targets for treatments of pancreatic fibrosis and cancer.

## Introduction

ATP is an extracellular signal that stimulates purinergic receptors in many different tissues. In pancreas ATP is released from acinar cells, pancreatic duct cells and from β-cells [1]–[3]. In 1998, a novel cell type was discovered in pancreas, namely the pancreatic stellate cell, PSC [4], [5]. The importance of the PSCs function in pancreas is becoming apparent, especially in the context of pancreatic disease such as chronic pancreatitis and pancreatic cancer [6]. Little is known about PSCs physiology and the role of purinergic signaling in these cells.

PSCs have a mixed phenotype and a protein expression profile overlapping with several different cell types. They express α smooth muscle actin (αSMA), which is typically expressed in fibroblasts that are able to contract, and glial fibrillary acidic protein (GFAP), an intermediate filament protein of astrocytes. These proteins are therefore not specific to PSCs, however, their combination, together with vitamin A rich lipid granules in freshly isolated cells, are specific markers for PSCs [4]. Similar stellate cells are found in many tissues in the body and the best characterized are the cells originating from the liver, named hepatic stellate cells [7].

In a healthy pancreas, PSCs are inactive and surround predominantly acinar cells. Only a few PSCs are found around ducts [8]. Upon pancreatic damage, metabolic stress and pancreatic cancer, PSCs become activated by growth factors/cytokines released from the neighboring cells [9], [10]. The activated PSCs then participate in wound healing. Subsequently, they either retreat via apoptosis or remain continuously activated. The latter scenario gives rise to pancreatic fibrosis [10], [11].

There are two main families of purinergic receptors for ATP: the P2Y receptor family of G-protein coupled receptors and the P2X receptor family of ligand-gated ion channels. The P2X receptors are annotated P2X1–P2X7 [12]. One of the most multifaceted receptors is the P2X7 receptor, which has a large intracellular C-terminal and forms a cation channel at micromolar ATP concentrations. At higher concentration of ATP, in the millimolar range, the receptor can open as a pore permeable to molecules up to 900 Da [13], [14]. This leads to apoptosis/necrosis, and therefore the receptor has been named the death receptor [15]–[17]. However, experiments by Baricordi *et al*. [18] indicated that the receptor has also proliferation determining properties when expressed in lymphoid cells. This behavior could occur at lower ATP concentrations and is most likely not due to pore-forming abilities of the receptor. The P2X7 receptor in rodents and humans exists in many different isoforms: in rodents the full length A (595 amino acids - aa) and K (592aa) isoforms with different starting exons, two C-terminally truncated versions named B (431aa) and C (442aa), and isoform D with only one transmembrane domain (153aa) [19]. Adinolfi *et al*. [20] suggested that the truncated isoform B stimulate proliferation in transfected HEK293 cells. Furthermore, expression of both A and B isoforms leads to additive effects on proliferation. A number of studies show that the P2X7 receptor stimulates a variety of cell responses and there are a number of SNPs that are associated with human disease [21], [22].

In pancreas, there are number of P2 receptors on exocrine and endocrine cells [1], [3]. In samples from patients suffering from chronic pancreatitis, or from mouse models of this disease, CD39, P2Y2 and P2X7 receptors were upregulated [23], [24]. However, the expression of the P2X7 receptor in PSCs is unsettled, as the experimental evidence is contradicting or incomplete; expression of the receptor was claimed by Künzli *et al*. but Hennigs *et al*. could not detect any expression [24], [25]. In another study, Won *et al*. [26] illustrated that ATP increases calcium signals in the nucleus of PSCs and this event was independent of extracellular calcium, implicating a P2Y type receptor.

The regulation of PSCs viability and function is highly relevant for many pancreatic diseases and we hypothesized that the P2X7 receptor could be an important element in this regulation. The aim of our experiments was to determine the expression of the P2X7 receptor and its effect on proliferation and/or death in freshly isolated PSCs. For this purpose we further developed and simplified the isolation method of PSCs that were obtained from WT and the Pfizer P2X7 receptor KO mice [27]. The experiments presented here show that the proliferation of PSC is dependent on the P2X7 receptor and on ATP concentrations, such that ATP can function as a promoter of proliferation at micromolar concentrations. At millimolar concentrations, however, ATP is lethal to PSCs.

## Methods

### Isolation of Cells

P2X7 KO and WT mice were bred from Pfizer (NOD.129P2(B6)-P2rx7^tm1Gab^/DvsJ) on the C57BL/6JBom background. Additional WT mice were purchased from Taconic (C57BL/6JBomTac). Mice of mixed gender weighing approximately 20 g were used in this study. For comparison experiments WT and KO mice were littermates. Procedures were approved by the Danish Animal Experiment Inspectorate (Dyreforsøgstilsynet). All isolation steps were carried out to minimize the risk of contamination. The pancreata from P2X7R (P2X7 Receptor) KO and WT mice were removed after the mice were killed with cervical dislocation. Connective tissue and fat were removed and the pancreas was cut into pieces in physiological Ringer. Isolation media contained DMEM and F12 (1∶1), 5 mM HEPES, 1 mg/ml Albumin, 2 mM CaCl_2_ and 5 mM Glycine, gassed with 5% CO_2_ and pH adjusted to 7.4. The small pieces were incubated in 5 ml of media containing 6 mg of Collagenase V (Sigma C9263), gassed with 5% CO_2_, 20% O_2_ and 75% N_2_ for 50 minutes at 37°C. Digested pieces were dispersed by vigorous pipetting with a glass pipette. The cell suspension was then centrifuged at 1000 g for 8 minutes. The media was changed and the cells were resuspended and added to a 10 cm plastic dish (NUNC) that was coated with 1 ml of FCS. After three hours, the dish was washed with media, and PSCs were the only cells that attached strongly. PSCs then appeared as small dark spots, some having small protrusions. The DMEM media (10% serum and Pen Strep, Invitrogen) was changed daily the first two days.

### Immunocytochemistry

PSCs were split and allowed to attach to glass coverslips overnight. The cells were fixed in 4% paraformaldehyde in PBS for 15 min, treated with 0.2 M TRIS-glycine (pH 7.4) for 15 min; then rinsed in PBS and permeabilized for 10 min in PBS 0.5% TritonX-100. The samples were blocked with 10% BSA in PBS for 45 minutes and then incubated with the primary antibodies for P2X7R (1∶100, 8 µg/ml, Alomone APR 004), αSMA (1∶400 Sigma A2547) or GFAP (1∶200 Sigma G9269) overnight at 4°C. The slides were then washed in PBS and incubated for 1 hour with 1∶400 dilution of appropriate secondary antibody conjugated to Alexa 488 or Alexa 568. For nuclear staining, DAPI was used (1∶400, 1 µg/ml). Fat droplets were stained with 5 mM Nile Red (Sigma 72485) in 75% glycerol for 15 minutes and washed. Finally, the coverslips were washed in distilled water and mounted with N-propyl-gallate on slides. Fluorescence was examined with 40× 1.3 NA objective in Leica (TCS SP 5X MP) confocal laser scanning microscope (Leica Microsystems A/S, Heidelberg).

### RNA Isolation and PCR

Cells were cultured to confluence and then RNA was isolated with RNeasy Mini Kit (Qiagen 74104 Briefly, cells lysates were precipitated with 1 volume of 70% ethanol and column purified. The RNA was treated with DNase 1 (RNase free DNase Set, Qiagen 79254). 300 ng of extracted RNA was used per reaction mixture in QIAGEN OneStep RT-PCR Kit (210212) analysis, with amplification parameters as follows: one cycle at 50°C for 30 min and one cycle at 95°C for 15 min followed by 40 cycles at 95°C for 30 s, 58°C for 30 s, 72°C for 30 s, and finally, one cycle at 72°C for 10 min. Subsequently, all transcripts were subjected to electrophoresis on 1.2% agarose gels. Table 1 shows primers used; these were synthesized by TAG Copenhagen A/S (Copenhagen, Denmark). All primers were designed using Primer-BLAST (NCBI) with an expected Tm of 60°C, annealing temperature was selected to be 2°C below this Tm.

**Table 1:** Primer sets used for RT-PCR on PSCs for isoforms A–D and K, and within the deleted area (Intra) and spanning the deleted area (Spanning).

Primers	Sequence	Product length
P2X7 A FW	TGCACTCTTGAGGAGCGCCGA	218
P2X7 A RW	TGATTCCTCCCTGAACTGCCACCT	
P2X7 B FW	GCCGCACATTCGCATGGTGG	247
P2X7 B RW	TGGCTGAATGGTTACGACCACTTGCT	
P2X7 C FW	TGCTTTCTGCAGGTCGGGGGT	228
P2X7 C RW	GAAACAAGTATCTAGGTTGGAACTTCTTGGCC	
P2X7 D FW	ACACCTTCCCTTTGCAGGGGAACT	197
P2X7 D RW	GGCAGCAGAACTTTAGGCCAGCT	
P2X7 K FW	GGATCGGGACGCTGAAGAACAC	173
P2X7 K RW	CTTCGTCACCCCACCCTCTGTGA	
P2X7 Intra FW	TCCAAGCTCTTCCATAAGCTCGTGC	70
P2X7 Intra FW	AGGGATCCTGGTAAAGCAGGAGGAG	
P2X7 Spanning FW	GCCCGAGTTGGTGCCAGTGT	223
P2X7 Spanning FW	TGTCGCAGCCTGCTGTTGGT	

### Western Blot

Protein lysates were created by adding 5× lysis buffer (250 mM TrisBase, 1.25 M NaCl, 50 mM EDTA, 5% Triton X-100, 20 mM NaF) to the PSCs. Cell lysates were centrifuged at 15,000 g for 15 min. Western blot samples were either not reduced or reduced by heating to 90°C in the presence of 50 mM DTT (dithiothreitol) for 10 minutes and run on precast gels from Invitrogen. The membrane was blocked overnight at 4°C in 0.5% milk powder and 1% BSA together with 0.1% Tween 20. Primary antibody for P2X7R (1∶200, 4 µg/ml, Extracellular; Alomone APR 004, 1∶100, 2 µg/ml, C-terminal; Santa Cruz sc-15200) was added in blocking buffer for 1 hour. The appropriate secondary antibody conjugated to horse-radish peroxidase (1∶2000) was added in blocking buffer for 1 hour. Enzyme substrate was added and blots were viewed on a Fusion FX Vilber Lourmat.

### Video Microscopy

Isolated cultured PSCs were seeded onto collagen coated culture flasks (0.031 mg/mL Laminin, 0.031 mg/ml Fibronectin and 0.18 mg/mL Collagen IV) and attached for 3 hours. Subsequently, the DMEM medium was exchanged with Ringer’s solution containing in mM: 122.5 NaCl, 5.4 KCl, 0.8 MgCl_2_, 1.2 CaCl_2_, 1 NaH_2_PO_4_ 5.5 Glucose and 10 HEPES. The culture flasks were placed in a heating chamber at 37°C of an inverted Axiovert 25 (Carl Zeiss). Cell images were recorded using video cameras (models XCST70CE and XC-77CE, Hamamatsu/Sony) and PCvision frame grabber boards (Hamamatsu, Herrsching). Acquisition of images was controlled by HiPic and WASABI software (Hamamatsu). PSCs were left to rest for 30 minutes, and then stimulated with 5 mM ATP. Images of cells were taken every two minutes for 10 hours. Images selection and videos were made using ImageJ.

### Calcium Signals

PSCs were seeded in Wilco dishes with optical glass bottom. Cells were incubated with 5 µM Fluo-4AM (Invitrogen) in 15 minutes. PSCs were pre-incubated with suramin (100 µM), az10606120 (10 µM) or vehicle solutions 30 min before the experiment. PSCs were stimulated with 50 µM 2'(3')-*O*-(4-Benzoylbenzoyl)adenosine-5'-triphosphate (BzATP), 50 µM ATP, and 5 µM ionomycin, sequentially. Fluo-4 was excited at 488 nm with an Argon laser and fluorescence was collected at 500–570 nm using a PL Apo 20× NA 0.7 objective on Leica confocal/multiphoton microscope. Cells were kept at 37°C in physiological solutions containing in mM: 140 NaCl, 1 MgCl_2_, 1.5 CaCl_2_, 0.4 KH_2_PO_4_, 1.6 K_2_HPO_4_, 10 Glucose and 10 HEPES. Image analysis was performed in LAS AF software with 5 single cell responses shown in the figure together with the collective response from circa 50 cells per preparation and evaluated by whole frame analysis. Fluo-4 intensity is given as fluorescence ratio at time t in relation to time 0 (Ft/F0); and in response to agonists as ΔFt/F0.

### Cell Death Assays

ATP (5 mM) was added to PSCs that had been seeded in Wilco glass dishes. After 5 hours, annexin V FITC conjugated antibody (Sigma APOAF) was added together with the dye propidium iodide for 10 minutes and pictures were taken using Leica SP 5X MP microscope. Annexin V was exited at 488 nm and emitted light collected at 500–570 nm; propidium iodide was excited at 488 nm and emission was collected at 600–700 nm sequentially. In one series of experiment, Caspase 3/7 activation (Promega, Caspase-Glo 3/7 assay) was measured according to the instructions of the manufacturer.

### Cell Proliferation

Cells were isolated and grown on plastic dishes for 7 days, which led to their activation. The activated PSCs were split and added to 96 well plates (COSTAR) at 5000 PSCs/well. After attachment for 24 hours in 10% serum, the media was changed to that containing the indicated concentration of serum together with ATP and/or other agonists/antagonists. The PSCs were then left undisturbed for 48 hours. During the last 4 hours, cells were incubated with the reagents from ROCHE (BrdU) or DOJINDO (Cell Counting Kit 8) and processed according to the manufacturer′s instructions and with appropriate controls. Chemiluminescence and absorbance were measured in Fluostar OPTIMA. All manual cell-counting was done using a counting chamber or dish area in the microscope. All measurements were performed in either duplicates or triplicates.

The cell counting kit was used in the experiments where proliferation over many days was monitored in living cells due to limited toxicity. BrdU incorporation that monitors the actual DNA synthesis requires that the cells are fixed. This method has higher sensitivity and most data shown in the result section were performed with the BrdU kit. Experiments with Cell Counting kit 8 were done in parallel to these and showed similar results in all experiments.

### Chemicals and Statistics

All chemicals/kits were purchased from Sigma-Aldrich unless otherwise states. The inhibitors A438079 and az10606120 were both purchased from TOCRIS Bioscience. Data are shown as means ± SEM, *n* denotes a number of experiments on cells isolated from different animals. Students paired t test was applied when comparing two samples from the same animal and *P*<0.05 was accepted as significant. For comparison of responses to various agonists Dunn’s test in one-way Analysis of Variance (ANOVA) on Ranks was used. Data were analyzed in Origin or Microsoft Excel.

## Results

### Characterization of PSC Preparation and Morphology

**Figure 1:**
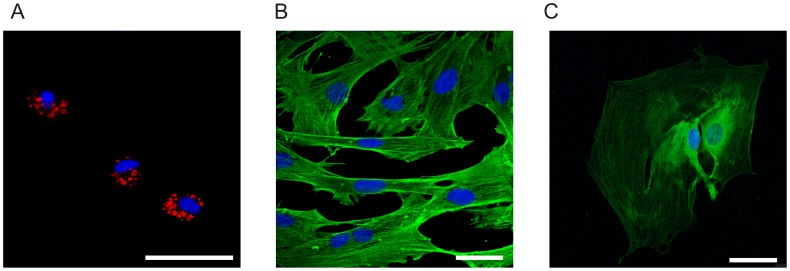
PSC phenotype. A. Nile red lipid staining at day 2. B. αSMA antibody staining at day 7. C. GFAP antibody staining at day 7. Secondary antibody was conjugated to Alexa 488 (green) and nuclear stain was DAPI (blue). Representative images of 3 independent experiments. Scale bars are 50 µm.

The two common methods for PSCs isolation make use of either a centrifugation gradients [4] or an outgrowth method [5]. The centrifugation method employs the low density of PSC in a Nycodense gradient to separate the cells. For the outgrowth method, pancreatic pieces are allowed to stay in media for longer time and PSCs migrate out of the tissue and proliferate. We aimed to simplify and scale down the isolation method for a single mouse pancreas. Therefore, we developed a method where PSCs were isolated using a combination of collagenase digestion and selective attachment. PSCs appear to have the ability to utilize the fibronectin in fetal calf serum for early attachment, as is the case for many cell types [28]. Therefore, PSCs attached quickly to the dish compared to ducts, acinar and islets cells, which did not attach to the dish in the first three hours. The stellate cell population obtained was uniform and showed clear lipid granules that could be detected up to day 2 (Fig. 1A). After 7 days, PSCs were activated and expressed clear α-SMA and also GFAP staining (Fig. 1B–C).

### The Expression of the P2X7 Receptor in PSCs

**Figure 2:**
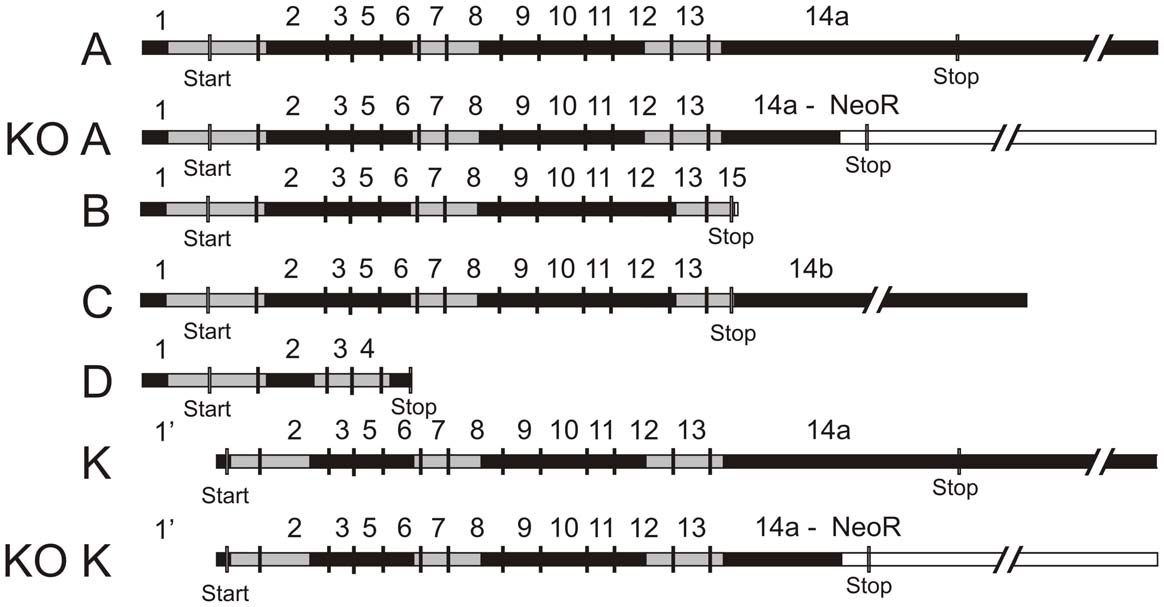
Gene map of the P2X7 receptor isoforms. The map is made according to the NCBI reference sequences. Exons are numbered and start/stop codon are marked. The map illustrates the potential mRNA isoforms that detected in mouse PSCs. These are marked in light grey for both WT and KO samples. Expected KO isoform A and K hybrids are also depicted, with the NeoR cassette inserted in white and the inserted early stop codon labeled.

**Figure 3:**
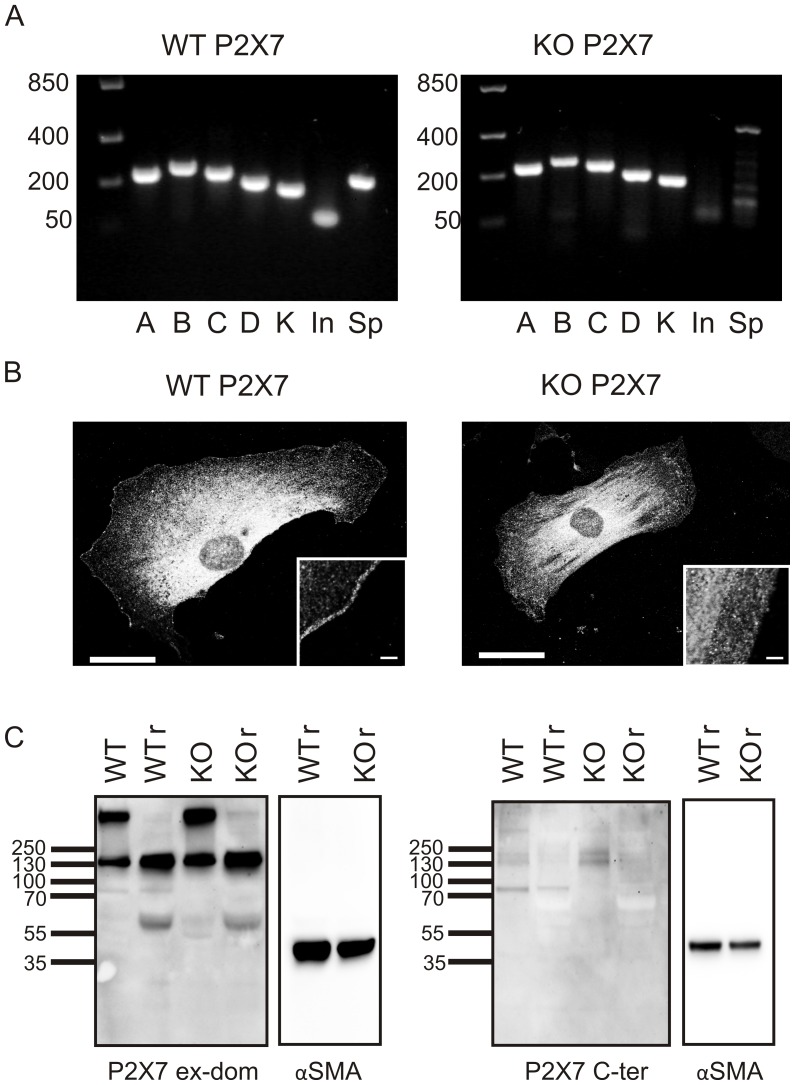
Expression of the P2X7 receptor in PSCs. A. PCR results from PSCs isolated from WT and KO mice illustrate the presence of all isoforms A–D and K; mRNA transcripts are also present in KO. The area disrupted by homologous recombination (In) is, however, absent. Similarly, the area spanning the disrupted area (Sp) is missing. B. Immunostaining of PSCs with primary antibody for the extracellular domain of the P2X7 receptor. Scale bar is 10 µM. C. Western blots on whole cell lysate with antibody against an extracellular domain (ex-dom) and the C-terminal part of P2X7 (C-term) in reducing (r) and non-reducing conditions. The loading control was αSMA and this blot was exposed for shorter times. Representative of 3 independent experiments.

We examined the P2X7 receptor expression using PCR, immunocytochemistry and Western Blot on WT and Pfizer KO samples. First, we investigated the mRNA content of the PSCs. The primer sets for PCR are given in Table 1. The creation of the Pfizer P2X7 receptor KO mice was made by inserting a Lox-NeoR-Promoter-Lox into the C-terminal instead of bp 1675 to bp 1760, this introduces an early stop codon truncating the C-terminal affecting isoforms A and K [27]. The map of isoforms and mRNA present in WT and KO is shown in Fig. 2. In the original paper, Solle *et al*. did not observe any mRNA in macrophages [27]. Fig. 3A shows that mRNA for all the mouse isoforms A–D and K are expressed WT PSCs. Surprisingly, in PSCs from Pfizer KO the mRNA for the exons downstream of the inserted neomycin box were still expressed, while the deleted area (P2X7R Intra) and the fragment amplified by primers spanning the insertion (P2X7R Spanning) were not present, as seen in the WT preparations. Nevertheless, the insertion of the NeoR part did lead to disruption of this part of the mRNA. Both the complete B and C isoform mRNAs of the P2X7 receptor are present in PSCs from KO (Fig. 3A). Also mRNA for the KO truncated P2X7 receptor isoform A (527aa) and K (524aa) with an early stop codon was detected (data not shown). This KO isoforms A and K hybrids were also reported by Masin *et al*. [29].

To determine whether any of the mRNA of the subtypes resulted in protein, we examined the protein expression using antibodies against the extracellular domain or the C-terminal part of the P2X7 receptor. Fig. 3B shows images of WT and KO PSCs labeled with the P2X7 receptor extracellular antibody, which labels intracellular vesicles and has a weaker but clear plasma membrane staining observed only in WT (see insert). The antibody against the C-terminus of the P2X7 receptor did not give any significant staining of the cells (data not shown). The same two antibodies were used for detecting the P2X7 receptor in a Western Blot in reducing and non-reducing conditions (Fig. 3C). Loading control (αSMA) was included for both blots. The expected size of a full length receptor monomer of the P2X7 receptor is 75 kDa and this is detected in WT with both antibodies (Fig. 3C, left and right panels). As seen in the figure, the extracellular antibody (left) also recognized a ∼450 kDa protein in non-reducing conditions in both WT and KO PSC samples; the band disappeared in the reduced sample and the 130 kDa band became more intense. These large proteins could be either P2X7 receptor trimers and/or a multiprotein complex, or a potential unspecific binding. Proteins of similar size have been reported by Kim *et al*. [30] and Masin *et al*. [29]. Importantly, the band for the shorter receptor isoform is expressed both in WT and KO and it is detected at about 60 kDa in reduced conditions only. This is most likely the isoform B or C. The C-terminal antibody (Fig. 3C, right) recognizes only the band at 75 kDa, demonstrating the full length P2X7 receptor monomer. Clearly, the C-terminus of P2X7 receptors is disrupted in KO PSCs and no protein was detected. Also the clear bands at 130 kDa and 450 kDa, which were sensitive to reducing conditions, were not obvious with the C-terminal antibody (Fig. 3C left and right panels).

### Differences in Cell Death in WT and KO PSCs

**Figure 4:**
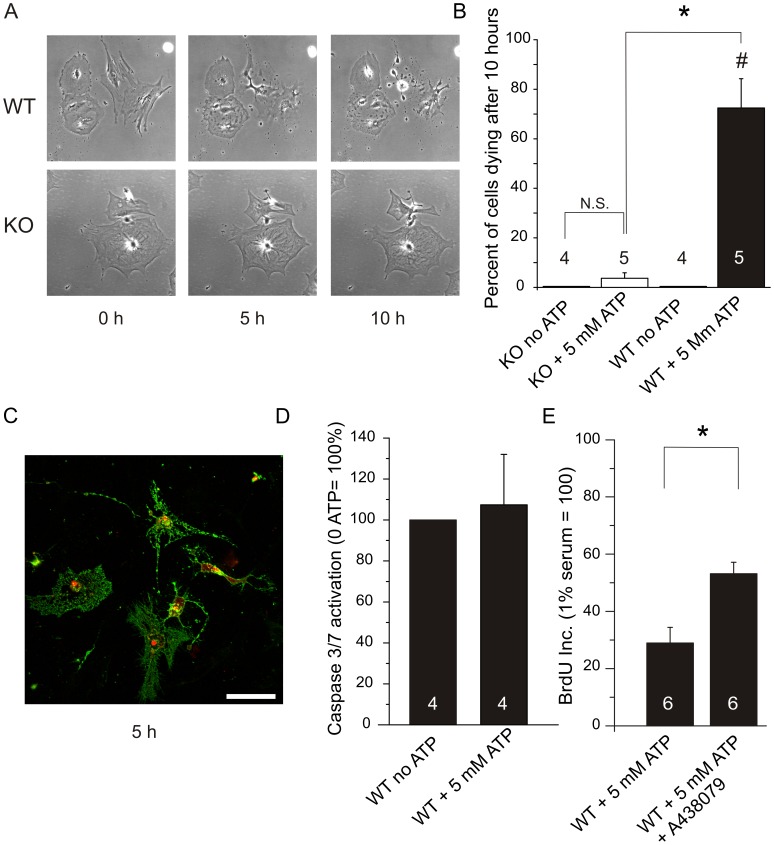
Cell death of PSCs in the presence of 5 mM ATP. A. Behavior of PSCs isolated from WT and KO was monitored for 10 hours; and pictures were taken at 2 min intervals. A. Representative phase contrast images of the PSCs at 0 hours, 5 hours and 10 hours. The top lane are WT cells and the bottom lane are KO cells. B. The bar graph summarizes the number of cells that died in control conditions or after addition of 5 mM ATP (n = 4–5 independent experiments). C. AnnexinV-FITC and propidium ioidide stain was added 5 hours after addition of 5 mM ATP to WT cells. D. Caspase 3/7 activation 5 hours after cells were stimulated with 5 mM ATP. E. BrdU incorporation measured 48 hours after the addition of 5 mM ATP. BrdU incorporations were normalized to 1% serum conditions. Significant difference (P<0.05) from respective controls (#) and between WT and KO (*) is indicated.

The mRNA data together with Western blot and immunocytochemistry illustrated that several isoforms of the P2X7 receptor are expressed in KO PSCs, including a truncated isoform K that escaped in the Glaxo KO [19]. However, this isoform must also be truncated in KO cells, as its last exon is the same as for isoform A. Therefore, we investigated whether the P2X7 receptor expressed in KO cells induced cell death, as is known to be the case for the full length A and K isoforms, presumably due to their pore-forming capabilities. Behavior of WT and KO PSCs was monitored by video microscopy in an environmentally controlled chamber for 10 hours. Without exogenous ATP, there was no significant death observed in the KO cells, nor in the WT PSC (Fig. 4A). The video capture in Fig. 4A shows that after the addition of 5 mM ATP, 72.5±12% (n = 5 independent experiments) of WT PSCs died within 10 hours compared with only 3.6±2% (n = 5) of KO PSCs. The data summarizing PSC death for WT and KO PSC is shown in Fig. 4B. These data clearly shows that the WT PSCs express functional P2X7 receptors that form cytolytic pores at 5 mM ATP. The P2X7 receptor protein that is expressed in KO PSCs, however, is not sufficient to cause pore opening and the following cell death. Since the cell death in WT cells visually appeared to have similarities with necrosis rather than apoptosis, we investigated which type of cell death might be initiated with high ATP concentrations. Using annexin-V FITC conjugated antibody together with propidium iodide, it is evident that the cells appear necrotic (Fig. 4C). In another assay we used apoptotic markers, caspase 3/7, and did not observe any significant activation after 5 hours (Fig. 4D), further supporting the notion that PSCs die by non-apoptotic, likely necrotic death. Importantly, the cell death that was initiated with 5 mM ATP could be inhibited by about 50% with the competitive P2X7 receptor-pore inhibitor A438079 at tested concentrations (Fig. 4E). These data show that the P2X7 receptor is important for initiating the cell death.

### Cell Numbers and Proliferation of PSCs

**Figure 5:**
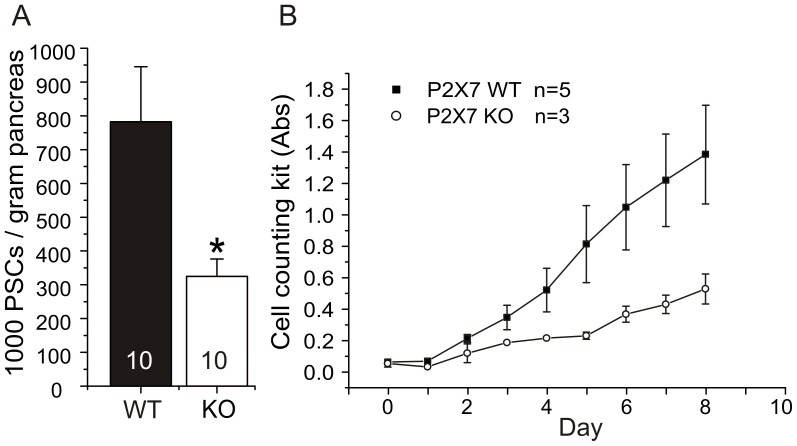
Differences in numbers of isolated and proliferating PSC cells. A. Number of isolated PSCs from pancreas of WT and KO mice (n = 10). PSCs were counted immediately after isolation in 5 separate areas of the dish. B. Difference in proliferation of WT and KO PSC following isolation and cell culture, as measured daily with Cell Counting Kit 8. (n = 3–5). Significant difference (P<0.05) between WT and KO (*) is indicated.

We compared the number of PSCs isolated from WT and KO animals. Fig. 5A shows that the number of PSCs isolated from KO was about 50% lower than the number of cells isolated from same amount of WT pancreas tissue (n = 10). Next we tested whether this difference in cell numbers is due to the differences in the proliferative potential of the PSC. The experiments were conducted to monitor cell proliferation rate *in vitro*. After isolation, PSCs from WT and KO animals were immediately split and re-seeded in 96 well plates with a density of 2500 cells/well. The cells were grown in 10% serum and the proliferation was determined with Cell Counting Kit 8 for a period of 8 days. The Fig. 5B shows that with the same seeding numbers, after first two days the growth of PSC from the KO mice lagged greatly behind the PSC from WT mice. The WT PSCs grew with a doubling time of 1.7 days and KO PSCs at 2.2 days, as calculated using the best fit exponential growth. Clearly, KO PSC had less proliferation potential. Since, there are several isoforms of the receptor expressed in KO PSCs (see Fig. 3), this finding indicates that the full length P2X7 receptor protein is required for the full proliferation effect.

The above experiments indicated that the P2X7 receptor could be important in regulating proliferation, therefore we postulated that by providing exogenous ATP we could further increase proliferation rate of PSCs. During the activation phase from day 0 to day 9, 100 µM ATP or the P2X7 receptor agonist 100 µM BzATP was included. The agonists were added to the cells daily when media was changed after Cell Counting Kit 8 measurements. However, addition of the agonists did not cause any difference in proliferation (data not shown). These experiments were carried out using 10% serum, so that effects of exogenously added ATP could have been masked by: (i) the stimulation of proliferation caused by the high serum; (ii) endogenous ATP release caused by daily medium changes; (iii) and not the least, changes in cells from inactive to active states. Therefore, we decided to simplify the protocols and work with activated PSC, lower serum concentrations and use the more sensitive BrdU kit. It became clear that as for many other cells, serum is important for PSC survival and proliferation. That is, 10% serum increased BrdU incorporation in PSCs to 386±51% (n = 4) compared to the 1% serum that was chosen as control for the following experiments.

### ATP Affects Both Life and Death of Activated PSCs

**Figure 6:**
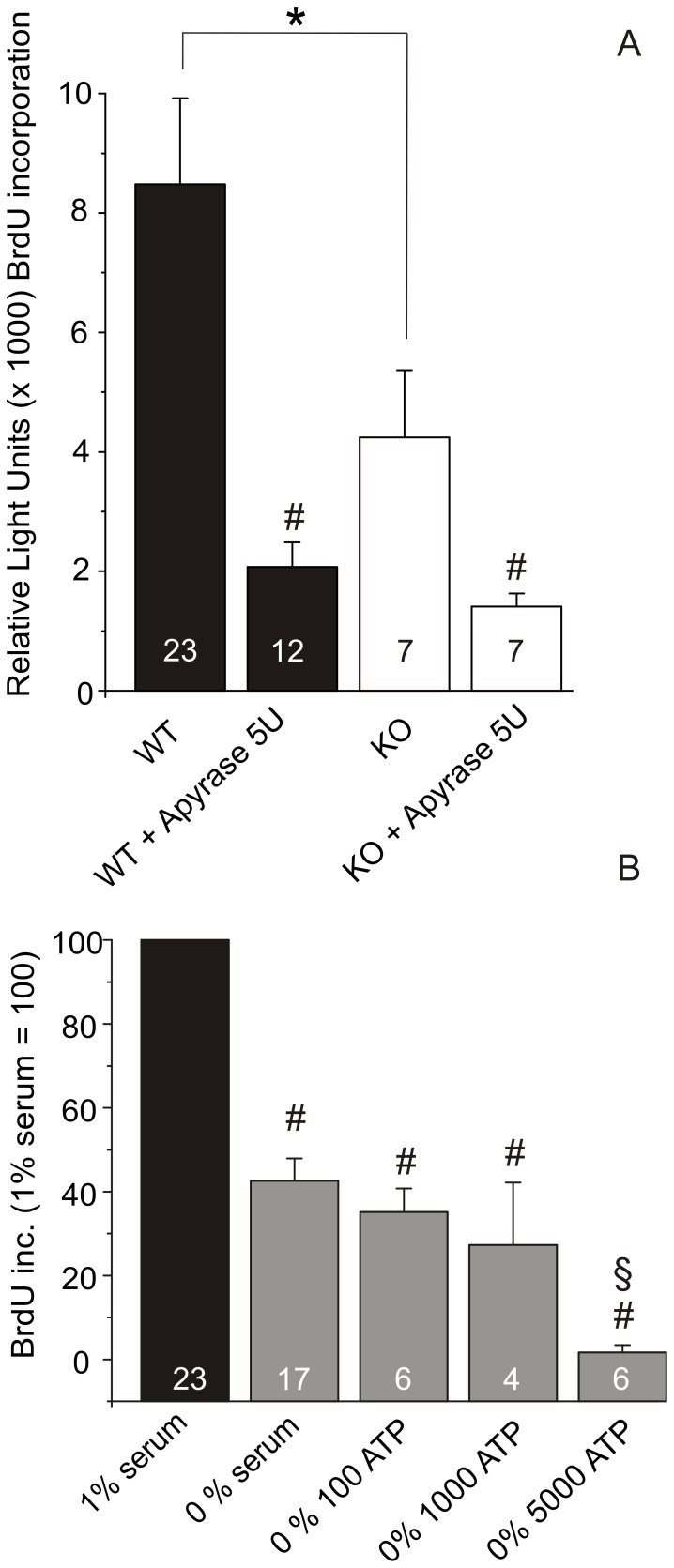
Effect of endogenous ATP and serum on PSCs proliferation. A. Comparison of the DNA incorporation in WT and KO PSCs with and without 5 U/ml apyrase. All samples contained 1% serum (n = 9–23). Y-axis shows the BrdU incorporation in relative light units. In the presence of apyrase, there was no significant difference between proliferation of WT and KO cells. Significant difference (P<0.05) from respective controls (#) and between WT and KO (*) is indicated. B. Effect of serum on DNA incorporation in WT PSCs. Significant difference (P<0.05) from 1% serum controls (#) and between 0% and 0% serum with 5 mM ATP (§) is indicated.

To investigate whether endogenous ATP and the P2X7 receptors were necessary for the proliferation of PSCs, we designed an experiment where cells were grown in the presence of apyrase, an enzyme that breaks down ATP/ADP to AMP. PSCs were grown in the presence of 1% serum, and proliferation of activated PSC cells (grown 7 days on plastic dishes) from WT and KO mice was determined using BrdU incorporation. Fig. 6A shows that KO PSCs proliferated significantly slower than WT cells, which confirmed the results obtained using different protocols (Fig. 5). In the presence of apyrase (5 U/ml), the proliferation rate was reduced in both WT and KO PSCs to similar levels. The cells did not die, but only stopped proliferating as indicated by measurements with the cell counting kit (data not shown).

We next tested whether ATP promotes proliferation in WT PSCs and whether it is independent of serum factors. All results were normalized to the proliferation rate with 1% serum set to 100%. Fig. 6B illustrates proliferation in 0% serum as BrdU incorporation was 42±5% (n = 17). For comparison, in 1% serum apyrase reduced BrdU incorporation to 24±4% (n = 12). Addition of exogenous ATP (100 µM) to serum-free medium did not change the proliferation rate of PSCs, i.e. BrdU incorporation was 35±6% (n = 6). However, addition of 5 mM ATP resulted in death of PSCs; BrdU incorporation was 2±2% (n = 6).

**Figure 7:**
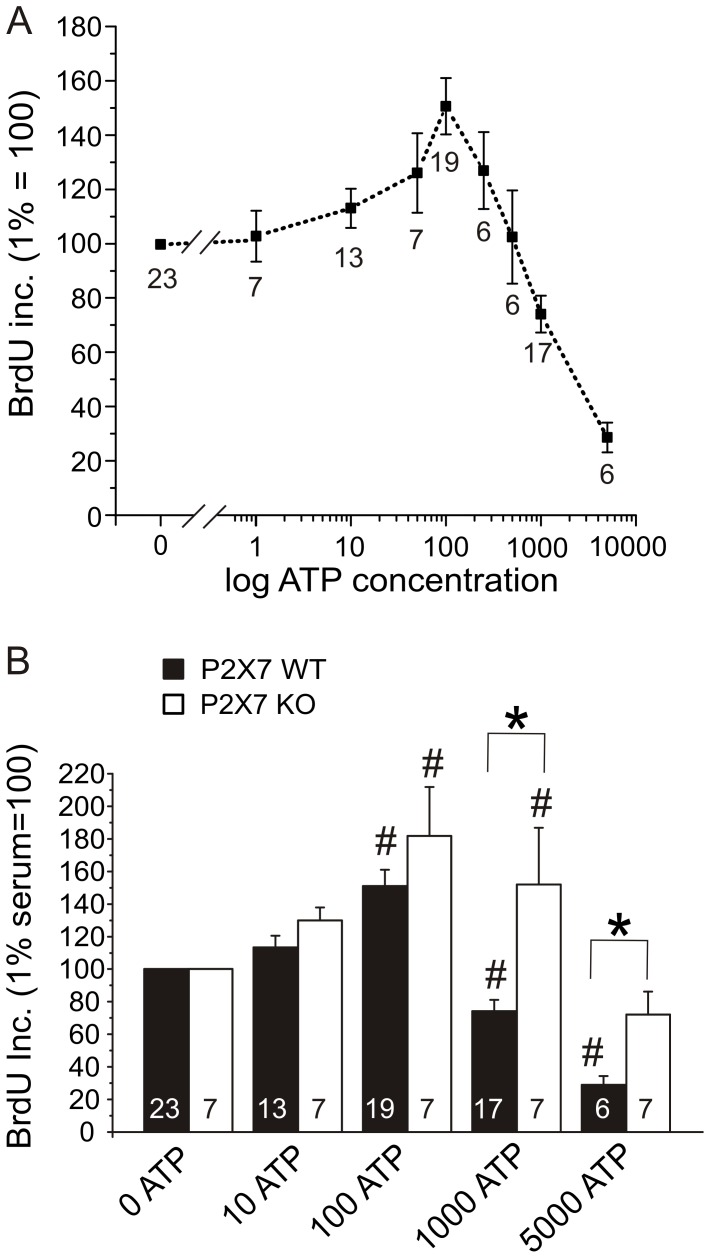
ATP can stimulate life and death in PSCs. A. Effect of ATP in growth medium with 1% serum on BrdU incorporation in WT PSCs (n = 6–23). B. Effect of selected ATP concentrations (µM) on BrdU incorporation in WT and KO PSCs both normalized to their respective 0 ATP controls (n = 7–23). BrdU incorporations were normalized to 1% serum conditions for the respective cell type. Significant difference (P<0.05) from respective controls (#) and between WT and KO (*) is indicated.

On the basis of the apyrase experiments, we concluded that endogenously released ATP could act as an autocrine proliferation potentiator for the activated PSCs in both WT and KO. We therefore hypothesized that exogenous ATP could further stimulate proliferation in 1% serum. Therefore, we tested the effect of increasing concentrations of ATP from 1 µM to 5 mM. The results shown in Fig. 7A were normalized to a 1% serum control without exogenous ATP. In WT PSCs, ATP stimulated proliferation in a concentration-dependent manner with a maximum effect at 100 µM (151±10%, n = 19). Higher concentrations of ATP did not stimulate proliferation, they actually lowered the DNA synthesis significantly at 1 mM (72±14%, n = 17). At 5 mM ATP, the DNA synthesis was greatly reduced (29±5%, n = 6) (Fig. 7A). Also the cell number was reduced, as confirmed by cell counting (data not shown).

In the following experiments, we determined the effect of ATP on BrdU incorporation in KO PSC and compared them to WT data. The raw value for WT PSC in 1% serum zero ATP control was 9839±1570 relative light units (RLU) and for KO PSCs it was 4596±1403 RLU (n = 23 and 7). Due to this difference between WT and KO, the controls were normalized so that ATP effects could be compared. As the results in Fig. 7B show, ATP stimulates proliferation in activated PSCs, both in WT and KO samples. This occurs in a concentration-dependent manner and 100 µM ATP gave the highest response in both types of cells with 151±10% increase for WT and 182±30% increase for KO, n = 19 and 17 (Fig. 7B). When the ATP concentration was increased further, there was a significant difference between proliferation of WT and KO PSCs. It is apparent that the KO PSCs did not die at high ATP concentrations of 1 mM, where their proliferation was 152±35% of the control compared to WT PSCs that had already reduced proliferation with values of 74±7% of the control (n = 7 and 17). At the highest ATP concentration tested (5 mM), there was a huge reduction of BrdU incorporation to 29±5% in WT PSCs, compared to 72±14% in KO PSCs, the latter being not significantly different to the control (n = 6 and 7).

**Figure 8:**
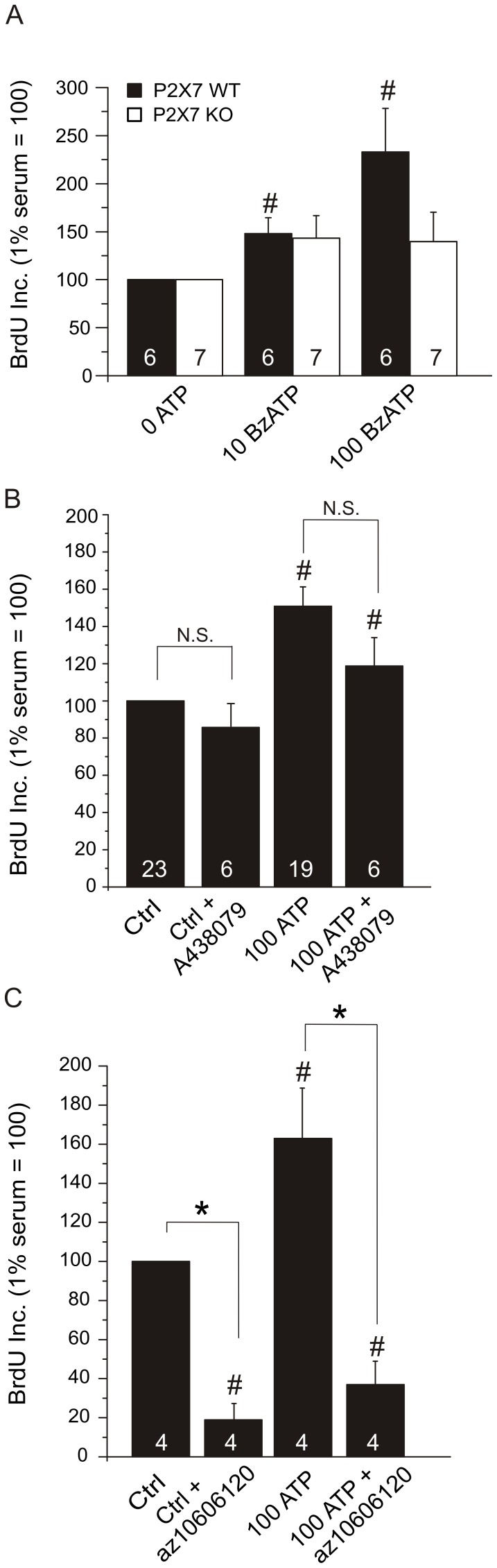
Effect of BzATP, A438079 and az10606120 on proliferation of PSC. A. The effect of BzATP on WT and KO PSCs (n = 6–7). All results were normalized to 1% serum controls that were set to 100%. The effect of: B. the P2X7 receptor A438079 inhibitor (10 µM); C. and negative allosteric modulator az10606120 (10 µM) on the basal proliferation response and in WT PSCs stimulated state with 100 µM ATP (n = 4–23). Significant difference (P<0.05) from respective controls (#) and with/without the inhibitor (*) is indicated.

Since the above experiments pointed towards the importance of the P2X7 receptor in life and death of PSCs, we postulated that a P2X7 receptor stimulant or inhibitor could affect the ATP stimulated proliferation. We therefore tested the effect of the prototypic P2X7 receptor agonist BzATP. Fig. 8A shows that BzATP increased the proliferation to 148±17% (n = 6) of the control at 10 µM and to 233±46% (n = 6) at 100 µM. Thus, 10 µM BzATP elicited a similar effect on cell proliferation as a tenfold higher ATP concentration. There also seemed to be a small increase of proliferation in KO PSCs at 10 µM BzATP (143±23%, n = 7) and at 100 µM (140±30%, n = 7), though no statistical significance was achieved. This observation could indicate that the P2X7 receptor isoforms expressed in KO cells do not respond well to BzATP.

The P2X7 receptor inhibitor A438079 is a competitive antagonist for P2X7 receptor specifically designed to prevent pore formation. When this antagonist (10 µM) was added 30 minutes before growth-stimulation by 100 µM ATP, or to PSCs growing in 1% serum, it did not show any significant effects (Fig. 8B). Therefore, we applied a non-competitive inhibitor, az10606120. This blocker binds in a positive cooperative manner to sites distinct from, but coupled to the ATP binding site and acts as a negative allosteric modulator [31]. Az10606120 (10 µM) was highly efficient in our experiments and inhibited the PSCs growth to 19±8% (n = 4) compared to 1% serum control (without exogenous ATP). The proliferative promoting effect of 100 µM ATP was also significantly reduced from 162±25% to 37±12% (n = 4) with this inhibitor.

**Figure 9:**
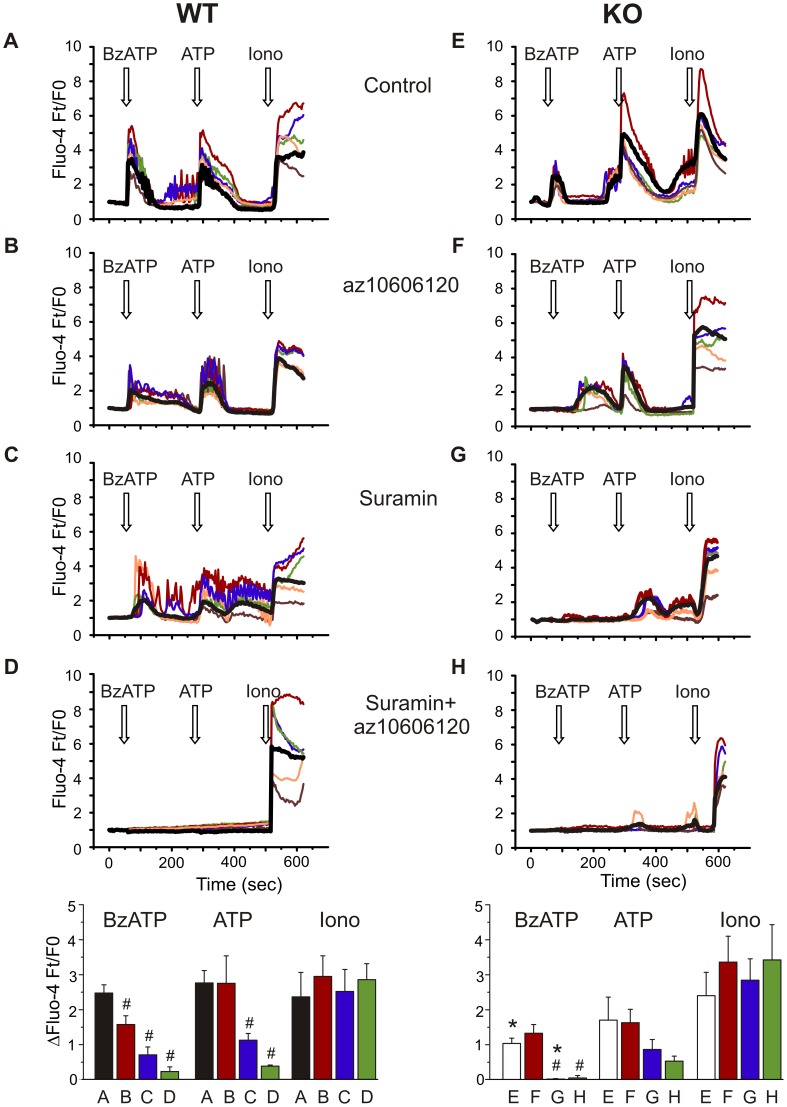
Calcium responses to BzATP and ATP in WT and KO PSC. The Fluo-4 response (Ft/F0) of WT (A–D) and KO (E–H) PSCs stimulated with 50 µM BzATP, 50 µM ATP and lastly with 5 µM ionomycin. Intracellular Ca^2+^ signals were monitored in control conditions (A, E), in presence az101606120 (B, F), suramin (C, G) and suramin together with az10606120 (D, H). The figures illustrate one independent representative run with the frame average for about 50 cells in black, and an example response of 5 individual cells is shown in color. The peak intracellular Ca^2+^ responses to agonists +/− antagonists derived from frame intensities for approximately 50 cells/preparation were averaged for 4 separate PSCs preparations form WT and KO mice and shown as bargraphs. The letters underneath the bars show from which experiment (indicated by a letter) the mean Ca^2+^ peak values were taken from. Significant difference (P<0.05) from respective controls (#) and in between WT and KO preparations (*) is indicated.

Finally, in order to obtain another evaluation of functional P2X7 receptor expression in WT and KO cells, calcium imaging was performed. Intracellular Ca^2+^ responses to BzATP (50 µM) and ATP (50 µM) and receptor inhibitors az10606120 (10 µM) and suramin (100 µM) were tested. Ionomycin (5 µM), a Ca^2+^ ionophore, was added in the end of the experiment as a positive control. Fig. 9 shows that there were significant differences in responses obtained from WT and KO cells. First of all, PSC from KO animals showed significantly smaller responses to BzATP. In WT cells BzATP changed the Fluo-4 intensity to 2.5±0.23 and az10606120 inhibitor (10 µM) lowered the response significantly to 1.6±0.25 (n = 4)(Fig. 9A, B). In KO cells, BzATP also elicited Ca^2+^ signals, Fluo-4 intensity increased to 1.0±0.15, and importantly, the az10606120 inhibitor had no further effects; i.e. Fluo-4 intensity was 1.3±0.24 (n = 4)(Fig. 9 E, F). This finding indicates that the az10606120 sensitive P2X7 receptor (isoform) remaining in PSCs from KO pancreas was not functional. In order to eliminate possible responses from other P2X and P2Y receptors, we added a broad spectrum inhibitor suramin at such a high concentration (100 µM) that most purinergic receptors would be blocked, and presumably only P2X7 responses would be visible (Fig. 9 C, G). Indeed, suramin inhibited the BzATP effect on Ca^2+^ signals completely in KO PSCs as Fluo-4 intensity remained at the baseline 0.0±0.02. In contrast, WT cells still had significant response (0.7±0.23, n = 4), and we postulate that this was due to P2X7 receptors. Combination of both az10606120 and suramin nearly eliminated Ca^2+^ responses to BzATP in PSCs from both WT (0.2±0.13, n = 4) and KO (0.0±0.07, n = 4) preparations. Regarding effects of the broader acting agonist ATP, Ca^2+^ responses appeared lower in KO compared to WT PSC (but not significant).

## Discussion

In the present study we have successfully isolated PSCs by a simplified method and studied their proliferation rate in response to purinergic signals. Activated PSCs grow faster in the presence of low concentrations of ATP. However, high ATP concentrations are cytotoxic to these cells. Solid evidence is provided for expression of the P2X7 receptors in PSCs. We propose that the P2X7 receptor isoforms are involved in regulation of PSC viability as discussed below.

PSCs isolated using a simplified method gave a substantial yield of PSCs from a single mouse pancreas. As shown in Fig. 1, PSCs were homogenous and had the same characteristics as determined in previous studies using established methods of gradient centrifugation [4] and outgrowth [5]. The homogenous expression of α-SMA, GFAP and lipid granules in these cells shows that this high-yield method of isolation is equivalent to established isolation methods.

We clearly show that PSCs express the P2X7 receptor on mRNA transcripts and protein level (Fig. 3B, C). All of the receptor isoforms (A-D and K) found in mice are expressed in the WT PSC (Fig. 3A). The KO PSCs also show mRNA for B–D isoforms and the C-terminally truncated A and K hybrid isoforms. Notably, PSCs from P2X7R KO mice also contained transcripts for the mRNA upstream relative to the 85 nucleobases in P2X7R that were replaced by the NeoR-plasmid insert. This expression profile of the P2X7 receptor is probably also relevant for other tissues. Our study indicates that the B (or C) isoform protein is expressed, as we detect a band at the expected size of 60 kDa in the Western blot with the extracellular antibody (Fig. 3C). This is highly relevant as the isoforms can be partially responsible for the proliferation effect in KO PSCs, and at least the B isoform has been implicated as a growth promoter [20]. In addition to the expressed B or C protein detected, we cannot determine the identity of the higher molecular weight bands. These could contain P2X7 protein coded by the transcript for either the isoforms KO A or K hybrids, B or C, as all of these versions will be recognized by the extracellular antibody. This is also the conclusion reached in a recent paper re-evaluating the P2X7 receptor expression in P2X7R KO mice by Masin *et al*. [29].

Interestingly, using immunocytochemistry we find that the P2X7 receptor antibody shows marked intracellular locations in both KO and WT PSC, which could be due to receptor desensitization during preparation. This has been seen regularly with P2X7R staining [32] and P2X7R-EGFP expression [33]. Intracellular hotspot staining is similar to that found for many other receptors, for example GABA [34]. Although this intracellular staining could be unspecific, we also see a clear membrane stain in the WT cells that is not observed in the KO cells (Fig. 3B). We also performed a functional assay of the P2X7 receptor using Ca^2+^ imaging (Fig. 9). Interestingly, there was still a response to BzATP in KO PSCs, but this could not be inhibited by the P2X7 receptor blocker az10606120 as it could for the WT PSCs. This supports the notion that the major P2X7 receptor isoform, possibly B or C, is not functioning as a cation channel in KO PSCs. The possible explanation for the BzATP effects in KO cells is that it could be mediated by other purinergic receptors, such as the P2X1 receptor that has affinity for this agonist [35], or that expressed isoforms are not sensitive to az10606120 inhibitor.

Several of our experiments show that the P2X7 receptor in WT PSCs is a death receptor when exposed to high ATP concentrations (Fig. 4, 7). However, PSCs from KO animals do not undergo cell death under the same conditions. This illustrates that the main receptor phenotype supposedly altered in the KO mice is correct and cells escape death. The protein site where the “death sequence” is located is most likely in the KO region (506aa-532aa) of the C-terminal. In this region, there is a sequence that is similar to the TNF-Receptor1 death domain (436aa-531aa) [36]. The KO receptor data in PSCs strengthen the prediction about the death region in the A and K isoform C-termini. Our experiments also indicate that cell death in PSCs might be of a necrotic character (Fig. 4 C, D), which has also been seen in other studies [15], [16].

One of the most important outcomes of our study is that the full length P2X7 receptor (A and/or K isoform) is important for optimal regulation of proliferation in PSCs. Firstly, *in vivo* PSCs isolated from KO mice were about 50% lower in numbers compared with cells isolated from the WT mice (Fig. 5A). This agrees with the study of Glas *et al*. [37], who found fewer pancreatic β-cells in the P2X7 receptor KO animals. Clearly, the lower number of cells from the KO tissue would not be expected if the main effect of the P2X7 receptor was that of a death receptor. Since the KO cells lack the full length P2X7 receptor, and there is a lower number of PSCs, we argue that the main property of the P2X7 receptor is to maintain proliferation of these cells in pancreas. Secondly, *in vitro* the KO PSCs grow much slower than WT PSCs as verified by several protocols (Fig. 5, 6).

Basal ATP release occurs in many cells [38]. In apyrase experiments we show that endogenous ATP is important for proliferation of PSC (Fig. 6A). Since this is the case for both WT and KO cells, one could infer that the isoforms expressed in KO PSCs, potentially the B or C variant detected, can partly compensate for the loss of potentiating effect of the full length P2X7 receptor (see below).

In order to simulate a stimulatory autocrine or paracrine release of ATP, exogenous ATP was added to PSCs. Most importantly, proliferation of PSCs was stimulated with ATP concentrations up to 100 µM (Fig. 7). We suggest that the basic proliferative response is mediated by either one of the truncated isoforms B or C, or potentially a KO A and K version, which has a truncated C-terminal. The N-terminal of the P2X7 receptor, which is still present in the KO, could transduce proliferative signaling via ERK1/ERK2 [33]. Nevertheless, for the full proliferative effects of exogenous and endogenous ATP seen in WT cells, the full length P2X7 receptor is required. Together, these are therefore the first experiments that illustrate the proliferation potential of the P2X7 isoforms in native cells. Our findings are consistent with the reports of Adinolfi *et al*. on HEK293 cells transfected with the P2X7 receptor [20] and Monif *et al*. on glial cells transfected with the P2X7 receptor [39]. Our PSCs do also require some serum for growth (Fig. 6B), perhaps because they are primary cells.

Both the proliferative and death effects of the P2X7 receptor in PSCs are supported by pharmacological data, which give new insights. BzATP stimulated proliferation in WT PSCs with about tenfold higher potency than ATP (Fig. 8A), implicating a P2X7 receptor effect. However, in KO PSCs, the proliferation effect of BzATP was small and also lower compared to ATP. Amstrup and Novak [33] illustrated that a ΔC from Ser365 showed smaller Ca^2+^ response to BzATP, but ATP effect was unchanged, which could indicate that the C-terminal is important for Ca^2+^ signaling transduction. The competitive blocker A438079 inhibited cell death in PSCs, but the effect on proliferation was marginal (Fig. 8B). The negative allosteric modulator az10606120, on the other hand, had significant effect on growth induced by micromolar ATP concentrations (Fig. 8C). These data agree well with the intracellular Ca^2+^ measurements showing that the az10606120 significantly inhibited response in WT PSCs (Fig. 9 C).

It is possible that the effect of ATP on proliferation could differ from the ATP effect on pore formation and cell death due to different binding sites on the receptor. In accordance, Klapperstück *et al*. [40] showed that there are two high sensitivity binding sites for ATP (4 µM) and two low sensitivity binding sites for ATP (200 µM) on the P2X7 receptor. The high sensitivity signal was dependent on both the N- and C-terminal for full transduction, while the low sensitivity only depended on the C-terminal. Therefore we propose, based on the KO data, that the high sensitivity site still can transduce some signal, and that BzATP might not be such a good ligand for this site. This is also confirmed by the Ca^2+^ imaging data, which showed significantly lower response to BzATP in KO compared with WT PSCs. We also conclude that the competitive inhibitor, A438079, is most likely binding to the low sensitivity site and might not be a good antagonist in respect to proliferation. The negative allosteric modulator, az10606120, has on the other and huge impact on the proliferation, suggesting that it modulates the high sensitivity site.

Based on the present *in vitro* experiments, extracellular ATP is an important proliferation regulator in PSCs. How can this be transferred to the *in vivo* situation? PSCs are located around the acinar cells [4], suggesting that their main function is related to their interplay with these cells. In a normal physiological situation acinar cells release zymogen granules containing digestive enzymes to the pancreatic duct lumen. In case of pancreatic damage, some zymogen granules could release their cargo the basolateral side of the acinus [41]. We have shown that zymogen granules accumulate ATP via VNUT/Slc17a9 transporter and thus contain ATP at concentrations that could activate purinergic receptors on surrounding cells [42]. Thus, when mild pancreatic damage occurs, we propose that PSCs proliferation that involves the P2X7 receptor would be stimulated to protect the damaged area. Hoque at al. [43] illustrated that P2X7 receptor KO mice, were less susceptible to develop maximal acute pancreatitis, however, they postulate that it was due to lack of expression of P2X7 on macrophages. The cytolytic effect of ATP is more difficult to explain. We propose that a massive damage and disruption of acinar cells and release of intracellular ATP (3–5 mM) can lead to the death of PSCs, presumably by “overstimulated” P2X7 receptors. The cytolytic P2X7 receptors stimulated in macrophages release IL-1β [27], [44]. It is a possibility that PSCs can convert the death signal of high ATP concentrations, into an interleukin release, which further activates and attracts other PSCs and immune cells. Whether the P2X7 receptor releases the interleukins, or whether it causes the cleavage of the propeptide, remains to be investigated.

### Conclusion

In conclusion, we show that basal and added ATP stimulated proliferation in PSCs. This effect is mediated by the P2X7 receptor, and maximal proliferation is obtained with 100 µM ATP. This effect and Ca^2+^ signaling could be inhibited by the specific P2X7 antagonist az10606120. We propose that the full proliferation effect requires the full length P2X7R isoform A or K. Nevertheless, since the KO PSCs show about half of the maximal proliferative response to endo/exogenous ATP, either KO A or K hybrid versions, or isoforms B or C are also involved. Since we find the expression of B or C variant proteins, we deem the latter more likely. At high ATP concentrations, exceeding 1 mM, the cytotoxic effect of ATP is dominating and the WT PSCs but not the KO cells die, and this could be partially inhibited by the antagonist A438079. Therefore, the C-terminal of the P2X7 receptor is necessary for the death signal.

Together this study presents strong evidence that ATP and the P2X7 receptors are important regulators of both life and death of PSCs, and therefore might be potential targets for treatments of pancreatic fibrosis and PSC interactions with other cells, for example, in the development of pancreatic ductal adenocarcinoma.
